# Real-world impact of antifibrotics on prognosis in patients with progressive fibrosing interstitial lung disease

**DOI:** 10.1136/rmdopen-2022-002667

**Published:** 2023-01-23

**Authors:** Takayuki Niitsu, Kiyoharu Fukushima, Sho Komukai, So Takata, Yuko Abe, Takuro Nii, Tomoki Kuge, Shinichi Iwakoshi, Takayuki Shiroyama, Kotaro Miyake, Kazuyuki Tujino, Satoshi Tanizaki, Kota Iwahori, Haruhiko Hirata, Keisuke Miki, Masahiro Yanagawa, Noriyuki Takeuchi, Yoshito Takeda, Hiroshi Kida, Atsushi Kumanogoh

**Affiliations:** 1 Respiratory Medicine and Clinical Immunology, Osaka University Faculty of Medicine Graduate School of Medicine, Suita, Osaka, Japan; 2 Respiratory Medicine, Osaka Toneyama Medical Center, Toyonaka, Osaka, Japan; 3 Biomedical Statistics, Osaka University Faculty of Medicine Graduate School of Medicine, Suita, Osaka, Japan; 4 Institute for Open and Transdisciplinary Research Initiatives, Osaka University, Suita, Osaka, Japan; 5 Rheumatology, Osaka Toneyama Medical Center, Toyonaka, Osaka, Japan; 6 Radiology, Nara Medical University, Kashihara, Nara, Japan; 7 Radiology, Osaka University Faculty of Medicine Graduate School of Medicine, Suita, Osaka, Japan; 8 Radiology, Osaka Toneyama Medical Center, Toyonaka, Osaka, Japan

**Keywords:** pulmonary fibrosis, therapeutics, fibroblasts

## Abstract

**Objective:**

No studies have demonstrated the real-world efficacy of antifibrotics for progressive fibrosing interstitial lung disease (PF-ILD). Therefore, we evaluated the efficacy of antifibrotics in patients with PF-ILD.

**Methods:**

We retrospectively reviewed the medical records of patients with ILD from January 2012 to July 2021. Patients were diagnosed with PF-ILD if they had ≥10% fibrosis on high-resolution CT (HRCT) and a relative forced vital capacity (FVC) decline of either ≥10% or >5% to <10% with clinical deterioration or progression of fibrosis on HRCT during overlapping windows of 2 years and with a %FVC of ≥45%. We compared FVC changes and overall survival (OS) between patients with and without antifibrotics. FVC changes were analysed using generalised estimating equations. We used inverse probability weighting (IPW) and statistical matching to adjust for covariates.

**Results:**

Of the 574 patients, 167 were diagnosed with PF-ILD (idiopathic pulmonary fibrosis (IPF), n=64; non-IPF, n=103). Antifibrotics improved the FVC decline in both IPF (p=0.002) and non-IPF (p=0.05) (IPW: IPF, p=0.015; non-IPF, p=0.031). Among patients with IPF, OS was longer in the antifibrotic group (log-rank p=0.001). However, among patients with non-IPF, OS was not longer in the antifibrotic group (p=0.3263) (IPW and statistical matching: IPF, p=0.0534 and p=0.0018; non-IPF, p=0.5663 and p=0.5618).

**Conclusion:**

This is the first real-world study to show that antifibrotics improve the FVC decline in PF-ILD. However, among patients with non-IPF, we found no significant difference in mortality between those with and without antifibrotics. Future studies must clarify whether antifibrotics improve the prognosis of non-IPF.

**WHAT IS ALREADY KNOWN ON THIS TOPIC**

- Recent studies have suggested that idiopathic pulmonary fibrosis (IPF) and progressive fibrosing interstitial lung disease (PF-ILD) share common fibrotic cascades that cause poor outcomes.
- In patients with IPF, the prognostic effect of antifibrotic agents has been well established. However, whether antifibrotic agents improve the forced vital capacity (FVC) decline or prolong overall survival in patients with PF-ILD in the real-world setting remains unclear. Hence, there is an unmet need for antifibrotics in patients with PF-ILD.

**WHAT THIS STUDY ADDS**

- The effect of antifibrotics differed between patients with IPF and those with non-IPF PF-ILD. In patients with non-IPF, antifibrotics alone were not significantly associated with mortality despite the fact that these agents suppressed the FVC decline.

**HOW THIS STUDY MIGHT AFFECT RESEARCH, PRACTICE OR POLICY**

- There is distinct validity regarding the effect of antifibrotics against FVC reduction and their use in clinical practice.
- However, improving the FVC might not be synonymous with improving the prognosis in the real-world management of PF-ILD.

## Introduction

Interstitial lung disease (ILD) is a heterogeneous group of disorders that include a variety of conditions, and it is a crucial complication in patients with connective tissue diseases (CTDs).^1^ Some patients with chronic ILD exhibit a progressive fibrosing phenotype called progressive fibrosing ILD (PF-ILD) or progressive pulmonary fibrosis,^2^ which has a prognosis as poor as that of idiopathic pulmonary fibrosis (IPF).^3^ This phenotype is accompanied by a temporal decline in lung function, increasing extent of fibrosis on high-resolution CT (HRCT) and worsening quality of life.^4–6^

The antifibrotic agent nintedanib was recently approved for the treatment of ILD with progressive fibrosis other than IPF based on the INBUILD trial, in which nintedanib demonstrated a reduction in lung function decline versus placebo.^7^ A recent subgroup analysis of the INBUILD trial suggested that nintedanib reduces the annual rate of decline in lung function regardless of the underlying ILD diagnosis.^8^

Today, ILDs are classified into those with and without progressive fibrosing phenotypes based on the disease progression.^2^ However, patients enrolled in clinical trials receive strictly controlled treatment for limited periods, which might not reflect the real-world situation. Moreover, the validity of recognising chronic ILD with a progressive phenotype as having a poor prognosis has been repeatedly shown in real-world cohort studies.^9^ ^10^ Although a real-world study on the efficacy of antifibrotics in patients with fibrosing phenotypes is urgently needed, no real-world studies have demonstrated the effectiveness of antifibrotics.

Therefore, we performed a multicentre retrospective cohort study to investigate the efficacy of antifibrotics for patients with PF-ILD in the real-world setting.

## Methods

### Ethics approval and consent to participate

This experimental protocol for data involving human participants followed the Ethical Guidelines of the Japan Ministries of Health and Labour for Medical and Health Research Involving Human Subjects. All experiments were conducted in accordance with the principles laid out in the Declaration of Helsinki.

### Study design and population

In this multicentre retrospective observational study, we reviewed the medical records of patients treated at Osaka University Hospital and the National Hospital Organization Osaka Toneyama Medical Centre from 1 January 2012 to 31 July 2021.

The data of patients diagnosed with chronic ILD on or after 1 January 2012 were extracted if the patients were ≥20 years old, had undergone at least three pulmonary function tests (PFTs) (at least one PFT was performed 6–18 months after the first test) and had received at least one HRCT examination. Based on the INBUILD trial,^7^ patients were considered to have a diagnosis of PF-ILD if they had ≥10% fibrosis on HRCT and met at least one of the following criteria for disease progression of ILD within the previous 24 months: relative decline in forced vital capacity (FVC) of ≥10% with or without clinical deterioration, relative decline in FVC between 5% and 10% associated with worsening respiratory symptoms, relative decline in FVC between 5% and 10% associated with an increased extent of fibrosis on chest HRCT or increased extent of fibrosis on chest HRCT with worsening respiratory symptoms. The date of diagnosis corresponded to the date on which the patient first met the criteria for PF-ILD. Patients were required to have an FVC of ≥45% of the predicted value. Patients lacking PFT results after the PF-ILD diagnosis were censored from the analysis of how antifibrotics affected lung function.

The HRCT scans were independently reviewed by two experienced thoracic radiologists (MY and NT) who were blinded to the patients’ clinical data. Furthermore, the medical records, laboratory and histological examination findings, bronchoalveolar lavage analysis results and HRCT findings were assessed by eight pulmonary physicians (Takayuki Niitsu, So Takata, YA, TK, TS, Satoshi Tanizaki, Kotaro Miyake and HH).

Based on these assessments and current guidelines and statements,^11–14^ the patients were diagnosed with IPF or non-IPF. Non-IPF included lung-dominant ILD (ldILD) (including idiopathic interstitial pneumonias (IIPs) other than IPF, hypersensitivity pneumonitis, sarcoidosis, silicosis, asbestosis and other conditions) and autoimmune ILD (CTD-ILD or interstitial pneumonia with autoimmune features).

CTD-ILD was diagnosed using a collaborative multidisciplinary approach with expert input from radiology, pathology, rheumatology (Takuro Nii and AK) and pulmonology specialists based on the EULAR/American College of Rheumatology classification criteria^15–19^ and the diagnostic criteria of the Japan Research Committee of the Ministry of Health, Labour and Welfare.^20^

Discrepancies were resolved through a consensus review performed by two experienced pulmonary physicians (KF and HK) and one thoracic radiologist (SI).

We collected data regarding age, sex, body mass index, chronic obstructive pulmonary disease, diabetes mellitus, blood monocyte count, lactate dehydrogenase concentration, sialylated carbohydrate antigen Krebs von den Lungen-6 concentration, C-reactive protein concentration, FVC, %FVC, diffusing capacity for carbon monoxide (DLCO), %DLCO and HRCT results (honeycombing, traction bronchiectasis) at the time of PF-ILD diagnosis and at the time of the first visit.

We also collected data regarding treatment modalities (antifibrotics (nintedanib and pirfenidone) and immunosuppressive agents (glucocorticoids (GCs) and immunosuppressants)) received at the time of PF-ILD diagnosis and at least once during the observation period.

These baseline characteristics were selected based on factors typically evaluated in patients with PF-ILD with the test closest to the date of PF-ILD diagnosis and the date of the first visit. We also defined the baseline FVC, %FVC, DLCO and %DLCO as the respiratory function test closest to the date of PF-ILD diagnosis (within 3 months before or after).

### Outcome measurements

To assess the effect of antifibrotic agents, the patients were classified into two groups: an antifibrotic group and a no-antifibrotic group. The antifibrotic group comprised patients who had taken antifibrotic drugs for at least three consecutive months from the date of diagnosis of PF-ILD until the last observation date. Regarding the choice of antifibrotic drugs, we defined the antifibrotic used as the first agent prescribed for three consecutive months.

To perform a rigorous statistical analysis, we defined the following preconfigured confounding factors based on previous reports of ILDs and PF-ILDs: age, sex, body mass index, differential diagnoses (IPF, autoimmune ILD and ldILD), HRCT findings of honeycombing, HRCT findings of traction bronchiectasis, FVC and GC therapy (prednisolone that had been received at a dosage of 10 mg/day for more than 1 month at the time of PF-ILD diagnosis).^21–30^

FVC measurements were attributed to time points every 6 months using a window of ±3 months; the nearest measurement was regarded as the specific time point for the analysis. Overall survival (OS) was defined as the time from PF-ILD diagnosis to death of any cause or the end of follow-up (end of observation period: 31 July 2021) with censoring at loss to follow-up or the end of the study period, whichever was later (for patients undergoing follow-up at other hospitals, information on the clinical course, including mortality, was constantly provided through the regional medical liaison office in most cases because both hospitals were the regional core specialised hospital). In the no-antifibrotic group, we also compared the annual rate of change in the %FVC from the time of PF-ILD diagnosis between patients with IIPs other than IPF and CTD-ILD using the Wilcoxon rank-sum test.

### Statistical analysis

We compared FVC changes and OS between the antifibrotic group and the no-antifibrotic group.

For comparison of the change in FVC between the antifibrotic group and no-antifibrotic group, we used generalised estimating equations (GEEs)^31 32^ to deal with repeated measures with the missing data from a single patient over time in generalised linear models. Patients lacking PFT results after the PF-ILD diagnosis were censored from the analysis of FVC changes.

To conduct the survival analyses, we applied the Kaplan-Meier method to estimate the OS in each treatment group and the log-rank test to compare the OS between groups.

To deal with missing data and to correct selection bias in this study, we used inverse probability weighting (IPW)^33^ with adjustment for the preconfigured covariates using propensity scores regarding FVC changes and OS.

All statistical analyses were performed using GraphPad Prism V.9 (GraphPad Software, San Diego, California, USA), JMP Pro V.16 (SAS Institute, Cary, North Carolina, USA), and R V.4.1.3 software. Continuous variables are reported as median and IQR. For continuous variables, Welch’s t-test and the Wilcoxon rank-sum test were used between two groups of independent samples based on the data distribution shown by the Shapiro-Wilk test. One-way analysis of variance and the Kruskal-Wallis test were used among three or more groups of independent samples based on the data distribution shown by the Shapiro-Wilk test. Additionally, the Steel-Dwass test for multiple comparisons was used for statistical analyses of three or more groups. For categorical variables, differences between groups were assessed using the χ2 test. When any cell had an expected count of <5, Fisher’s exact test was used instead of the χ2 test.

## Results

### Study population and baseline characteristics

**Figure 1:**
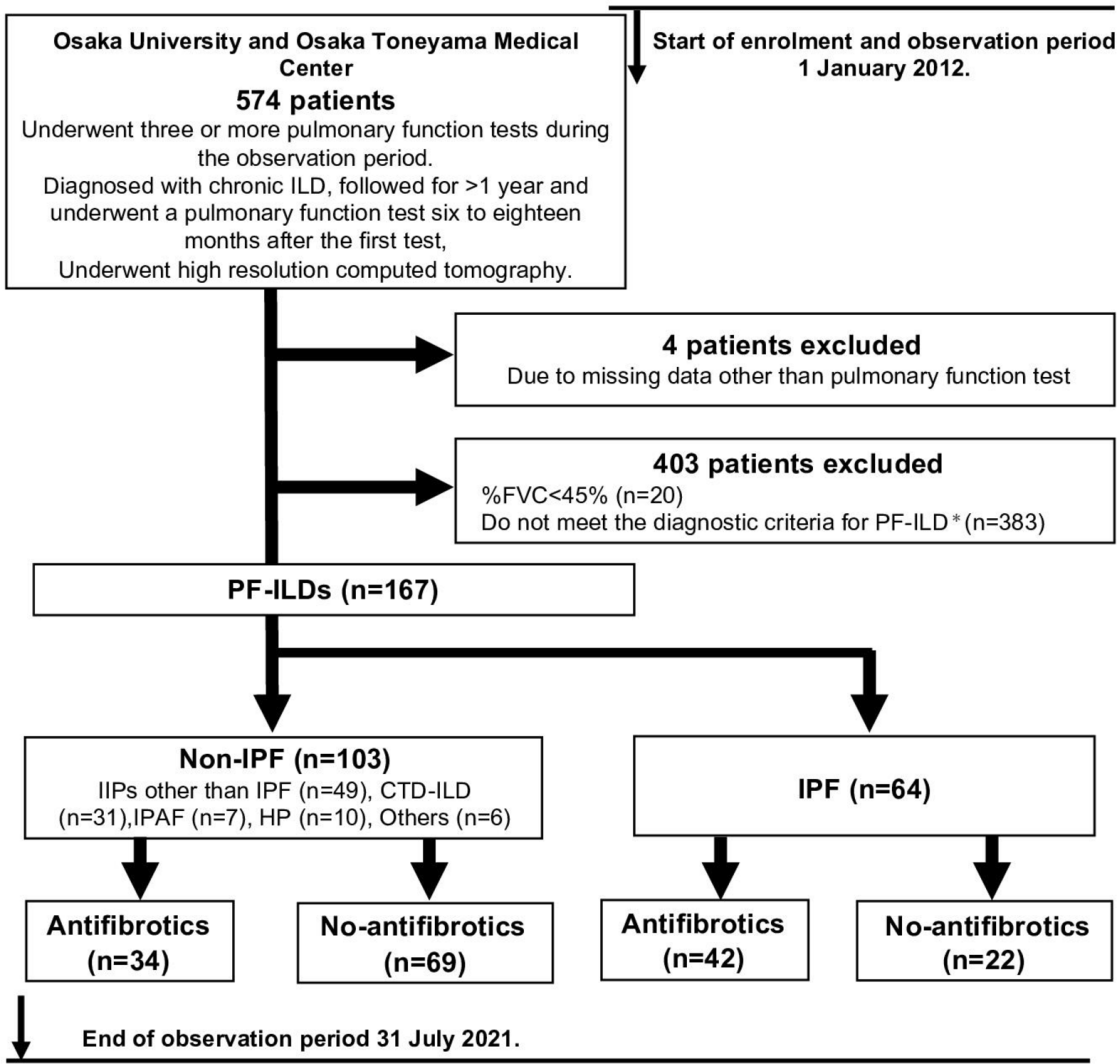
Study design and patient population. *Patients were diagnosed with PF-ILD if they had ≥10% fibrosis on high-resolution CT (HRCT) and a relative forced vital capacity (FVC) decline of either ≥10% or >5% to <10% with clinical deterioration or progression of fibrosis on HRCT during overlapping windows of 2 years. CTD-ILD, connective tissue disease-related interstitial lung disease; HP, hypersensitivity pneumonitis; IIP, idiopathic interstitial pneumonia; IPAF, interstitial pneumonia with autoimmune features; IPF, idiopathic pulmonary fibrosis; PF-ILD, progressive fibrosing interstitial lung disease.

During the enrolment period (1 January 2012 through 31 July 2021), 574 consecutive patients were diagnosed with ILD and followed for >1 year (until their last visit, death or the end of the observation period, as described in the Methods), during which time they underwent examination by HRCT and at least three PFTs (at least one PFT was performed 6–18 months after the first test) (figure 1).

Among the 574 patients, 407 were excluded because their %FVC was <45% (n=20), they did not meet the diagnostic criteria for PF-ILD (non-PF-ILD) (n=383), or they had missing data other than PFT and HRCT findings (n=4). The final cohort comprised of 167 patients with PF-ILD. Among these 167 patients, 64 were diagnosed with IPF and 103 were diagnosed with non-IPF. Among the patients with IPF, 42 were assigned to the antifibrotic group and 22 were assigned to the no-antifibrotic group (details are shown in online supplemental table S1). Among the patients with non-IPF, 34 were assigned to the antifibrotic group and 69 were assigned to the no-antifibrotic group (figure 1). Twelve patients were excluded from the analysis of FVC changes because they lacked PFT results after the PF-ILD diagnosis. In total, 155 patients were incorporated into this analysis (IPF, n=61; non-IPF, n=94).

10.1136/rmdopen-2022-002667.supp1 Supplementary data

The baseline characteristics of all included patients (combined and stratified according to diagnosis) are summarised in table 1 (IIPs other than IPF, n=49; CTD-ILD, n=31; interstitial pneumonia with autoimmune features, n=7; hypersensitivity pneumonitis, n=10; and others, n=6).

**Table 1:** Baseline characteristics and treatment modalities of patients with PF-ILD at PF-ILD diagnosis

		PF-ILD						P value
	Total (n=167)	IPF (n=64)	Non-IPF (n=103)					
			IIPs other than IPF (n=49), NSIP (n=19), PPFE (n=2), unclassified (n=26), COP (n=2)	CTD-ILD (n=31), SSc (n=12), RA (n=8), IM* (n=7), others† (n=4)	IPAF (n=7)	HP (n=10)	Others‡ (n=6)	
Characteristics								
Sex, male	115 (68.86) §	48 (75.0)	34 (69.39)	17 (54.84)	5 (71.43)	6 (60.0)	5 (83.33)	0.4330
Age (years)	71.0 (64.0–75.0)	73.0 (68.25–77.0)	70.0 (63.5–74.5)	64.0 (52.0–71.0)	73.0 (67.0–74.0)	69.5 (63.75–74.5)	71.5 (66.75–79.5)	0.0009
BMI	22.75 (21.2–25.33)	23.65 (21.77–26.16)	21.93 (20.17–24.41)	22.34 (20.7–25.11)	22.84 (22.02–24.26)	22.02 (20.98–25.56)	22.29 (19.57–28.32)	0.3754
COPD	24 (14.37)	9 (14.06)	8 (16.33)	3 (9.68)	0 (0.00)	2 (20.00)	2 (33.33)	0.4411
DM	24 (14.37)	11 (17.19)	7 (14.29)	4 (12.9)	1 (14.29)	0 (0.00)	1 (16.67)	0.5331
Serological examination								
Monocyte count	429.2 (331–545.8)	411.9 (331.8–531.8)	421.1 (312–556.2)	432.3 (335.8–712.5)	516.6 (354.5–557.4)	408.3 (265.5–480.3)	507.1 (417.2–610.2)	0.4060
LDH	217 (191.8–251.3)	215 (192-260)	218 (188-258)	233 (190-254)	221 (187-232)	213 (192.5–233)	217 (203.5–239)	0.9941
KL-6	896 (584.8–1357.2)	926.6 (681.5–1588.1)	791.5 (479.8–1079.9)	952.3 (618.3–1335)	826 (450.5–1503)	1425.2 (572.3–1898.5)	562.5 (336.8–806)	0.0037
CRP	0.16 (0.1–0.38)	0.16 (0.1–0.44)	0.11 (0.08–0.11)	0.31 (0.11–0.73)	0.1 (0.05–0.77)	0.18 (0.11–0.29)	0.18 (0.13–0.35)	0.2253
Pulmonary function								
FVC	2.29 (1.8–2.84)	2.32 (1.90–2.85)	2.42 (1.85–2.86)	2.19 (1.7–2.61)	2.39 (1.79–3.48)	1.99 (1.67–2.6)	3.01 (1.93–3.47)	0.5002
%FVC	82.2 (69.1–91.7)	84.45 (72.45–93.63)	79.5 (65.55–92)	78.3 (63.9–88.6)	87.9 (73.9–98.6)	72.15 (63.88–92.38)	81.2 (59.9–90.98)	0.1520
DLCO (n=125)	10.07 (7.47–12.0)	10.36 (7.88–12.20)	10.16 (7.25–12.27)	9.22 (6.36–11.74)	11.06 (7.21–13.38)	10.54 (7.74–12.66)	7.33 (5.62–8.91)	0.4235
%DLCO (n=125)	58.5 (44.6–75.27)	60.42 (47.03–78.09)	61.8 (43.5–80.99)	54.2 (34.55–62.85)	65.47 (50.65–76.30)	62.37 (51.37–74.68)	41 (39.4–49.18)	0.2098
Radiological findings								
Honeycombing	65 (38.92)	36 (56.25)	15 (30.61)	9 (29.03)	2 (28.57)	3 (30.0)	0 (0.0)	0.0084
Traction bronchiectasis	101 (60.48)	41 (64.06)	29 (59.18)	21 (67.74)	3 (42.86)	4 (40.0)	3 (50.0)	0.5361
Immunosuppressive agents								
Glucocorticoids (PSL<10 mg/day)	12 (5.99)	0 (0.0)	1 (1.96)	11 (35.48)	0 (0.0)	0 (0.0)	0 (0.0)	<0.001
Glucocorticoids (PSL≥10 mg/day)	23 (13.77)	7 (10.94)	7 (14.29)	7 (22.58)	0 (0.0)	2 (20.0)	0 (0.0)	0.5147
Immunosuppressant	16 (9.58)	2 (3.13)	5 (10.2)	9 (29.03)	0 (0.0)	0 (0.0)	0 (0.0)	0.0077

*Dermatomyositis (n=4), polymyositis (n=3).

†Microscopic polyangiitis (n=2), mixed connective tissue disease (n=1), systemic lupus erythematosus (n=1).

‡Exposure-related ILD (n=5), sarcoidosis (n=1)

§Data are shown as n (%) or median (IQR).

COPD; chronic obstructive lung pulmonary disease; BMI, body mass index; COP, cryptogenic organising pneumonia; CRP, C-reactive protein; CTD-ILD, connective tissue disease-interstitial lung disease; DLCO, diffusing capacity for carbon monoxide; DM, diabetes mellitus; FVC, forced vital capacity; HP, hypersensitivity pneumonitis; IIPs, idiopathic interstitial pneumonias; IM, inflammatory myopathies; IPAF, interstitial pneumonia with autoimmune features; IPF, idiopathic pulmonary fibrosis; KL-6, sialylated carbohydrate antigen Krebs von den Lungen-6; LDH, lactate dehydrogenase; NSIP, non-specific interstitial pneumonia; PF-ILD, progressive fibrosing interstitial lung disease; PPFE, pleuroparenchymal fibroelastosis; PSL, prednisolone; RA, rheumatoid arthritis; SSc, systemic sclerosis.

Among the patients with non-IPF PF-ILD, those with IIPs other than IPF were classified into patients with non-specific interstitial pneumonia (n=19), pleuroparenchymal fibroelastosis (n=2), cryptogenic organising pneumonia (n=2) and unclassifiable IIP (n=26). The patients with CTD-ILD were classified into those with systemic sclerosis (n=12), rheumatoid arthritis (n=8), inflammatory myopathies (dermatomyositis (n=4) and polymyositis (n=3)), microscopic polyangiitis (n=2), mixed CTD (n=1) and systemic lupus erythematosus (n=1). Although the patients with IPF were older and had a higher incidence of honeycombing, there was no other significant difference in the baseline characteristics between the disease groups other than sialylated carbohydrate antigen Krebs von den Lungen-6 and immunosuppressant use.

Table 2 compares the baseline characteristics between the antifibrotic group and no-antifibrotic group in both patients with IPF and those with non-IPF at the time of PF-ILD diagnosis.

**Table 2:** Baseline characteristics of patients with PF-ILD between the antifibrotic and no antifibrotic group at PF-ILD diagnosis

	PF-ILD							
	IPF				Non-IPF			
	Total (n=64)	Antifibrotic (n=42)	No-antifibrotic (n=22)	P value	Total (n=103)	Antifibrotic (n=34)	No-antifibrotic (n=69)	P value
Characteristics								
Sex, male	48 (75.0) *	29 (69.05)	19 (86.36)	0.2233	67 (65.05)	24 (70.59)	43 (62.32)	0.5112
Age (years)	73.0 (68.25–77.0)	71.5 (66.75 76.25)	74.5 (70.75–77.25)	0.1902	70.0 (62.0–74.0)	69.5 (63.25–74)	70 (61.0–73.5)	0.5672
BMI	23.65 (21.77–26.16)	23.81 (21.63–26.71)	23.27 (21.82–25.11)	0.7132	22.37 (20.44–25.07)	23.57 (20.98–26.47)	21.93 (20.02–24.26)	0.0198
COPD	9 (14.06)	5 (11.9)	4 (18.18)	0.4804	15 (12.62)	7 (20.59)	8 (11.59)	0.2452
DM	11 (17.19)	5 (11.9)	6 (27.27)	0.1659	13 (12.62)	3 (8.82)	10 (14.49)	0.5371
Serological examination								
Monocyte count	411.9 (331.8–531.8)	402.6 (329.7–539)	438.1 (332.3–515.1)	0.9464	432.2 (329.5–555.2)	437.7 (379.8–554.7)	427 (302.1–556.2)	0.387
LDH	215 (192–260)	214 (192–248)	216 (185.3–267.5)	0.9107	218 (190–251)	213 (181.5–246.5)	224.5 (190.8–251)	0.528
KL-6	926.6 (681.5–1588.1)	916.4 (740.5–1606)	1014.5 (538.9–1549.3)	0.8550	827.5 (571–1267.5)	829 (598.6–1423.1)	826 (513.8–1166.6)	0.369
CRP	0.16 (0.1–0.44)	0.16 (0.1–0.32)	0.155 (0.1–0.46)	0.9437	0.16 (0.1–0.36)	0.18 (0.1–0.35)	0.19 (0.1–0.42)	0.8083
Pulmonary function								
FVC	2.32 (1.90–2.85)	2.29 (1.89–2.87)	2.43 (1.88–2.86)	0.9039	2.28 (1.78–2.84)	2.33 (1.96–2.77)	2.24 ( 1.73–2.86)	0.9841
%FVC	84.45 (72.45–93.63)	86.55 (72.83–94)	84.2 (70.33–92.83)	0.5456	78.7 (65.1–90.4)	79.3 (63.0–90.75)	78.3 (67.2–89.3)	0.7737
DLCO (n=125)	10.36 (7.88–12.2)	11.04 (8.41–12.74)	8.61 (7.10–11.59)	0.0962	9.89 (7.29–11.87)	9.69 (7.26–11.76)	10.18 (7.37–12.22)	0.7664
%DLCO (n=125)	60.42 (47.03–78.09)	69.66 (49.05–80.97)	51.7 (42.4–69.72)	0.0527	57.8 (41.68–73.25)	52.8 (40.03–71.75)	61.05 (43.73–74.68)	0.3081
Radiological findings								
Honeycombing	36 (56.25)	22 (52.38)	14 (63.64)	0.4363	29 (28.16)	12 (35.29)	17 (24.64)	0.2581
Traction bronchiectasis	41 (64.06)	27 (64.29)	14 (63.63)	1.000	60 (58.25)	22 (64.71)	38 (55.07)	0.3512
Immunosuppressive agents								
Glucocorticoids (PSL<10 mg/day)	0 (0.0)	0 (0.0)	0 (0.0)	1.000	12 (11.65)	3 (8.82)	9 (13.04)	0.7464
Glucocorticoids (PSL≥10 mg/day)	7 (10.94)	4 (9.52)	3 (13.64)	0.6837	16 (15.53)	5 (14.71)	11 (15.94)	1.000
Immunosuppressant	2 (3.13)	1 (2.38)	1 (4.55)	1.000	14 (13.59)	6 (17.65)	8 (11.59)	0.5417

*Data are shown as n (%) or median (IQR).

COPD; chronic obstructive lung pulmonary disease; BMI, body mass index; CRP, C-reactive protein; DLCO, diffusing capacity for carbon monoxide; DM, diabetes mellitus; FVC, forced vital capacity; IPF, idiopathic pulmonary fibrosis; KL-6, sialylated carbohydrate antigen Krebs von den Lungen-6; LDH, lactate dehydrogenase; PF-ILD, progressive fibrosing interstitial lung disease; PSL, prednisolone.

Among patients with IPF, although not statistically significant, those in the antifibrotic group had higher DLCO and %DLCO than those in the no-antifibrotic group. Whereas there was no significant difference in FVC and %FVC. Among patients with non-IPF, those in the antifibrotic group had a higher body mass index than those in the no-antifibrotic group. There was no significant difference in the other variables. Regarding treatment modalities, there was no difference between the antifibrotic group and no-antifibrotic group in either IPF or non-IPF at the time of PF-ILD diagnosis. However, the frequency of immunosuppressive agent use increased in both patients with IPF and those with non-IPF during the PF-ILD disease course (online supplemental table S2). Patients with CTD-ILD more frequently received GCs (CTD-ILD, 24/31 (77.42%); IIPs other than IPF, 26/49 (53.06%)) and immunosuppressants (cyclosporine A, n=5; mycophenolate mofetil, n=7; cyclophosphamide, n=3; tocilizumab, n=3) (details are provided in online supplemental tables S3 and S4). All immunosuppressants for the patients with CTD-ILD were prescribed by rheumatologists.

In this study, we excluded a certain number of patients before the PF-ILD diagnosis. Thus, we compared the baseline characteristics between the excluded patients and patients with PF-ILD at the first visit (online supplemental table S5). Patients with a low %FVC were characterised by younger age, higher lactate dehydrogenase and sialylated carbohydrate antigen Krebs von den Lungen-6 concentrations and lower respiratory function. We also evaluated the difference between patients with PF-ILD (n=167) and patients with non-PF-ILD (n=383). Furthermore, the longitudinal change in respiratory function and the survival curve analysis from the time of the first visit to the last follow-up or death confirmed distinct characteristics between the two groups (online supplemental figure S1A, B and S2).

### Lung function evaluation

In this PF-ILD cohort, 155 patients underwent PFTs after PF-ILD diagnosis, and incorporated into lung function evaluation analysis of antifibrotics. Details are shown in online supplemental table S6.

**Figure 2:**
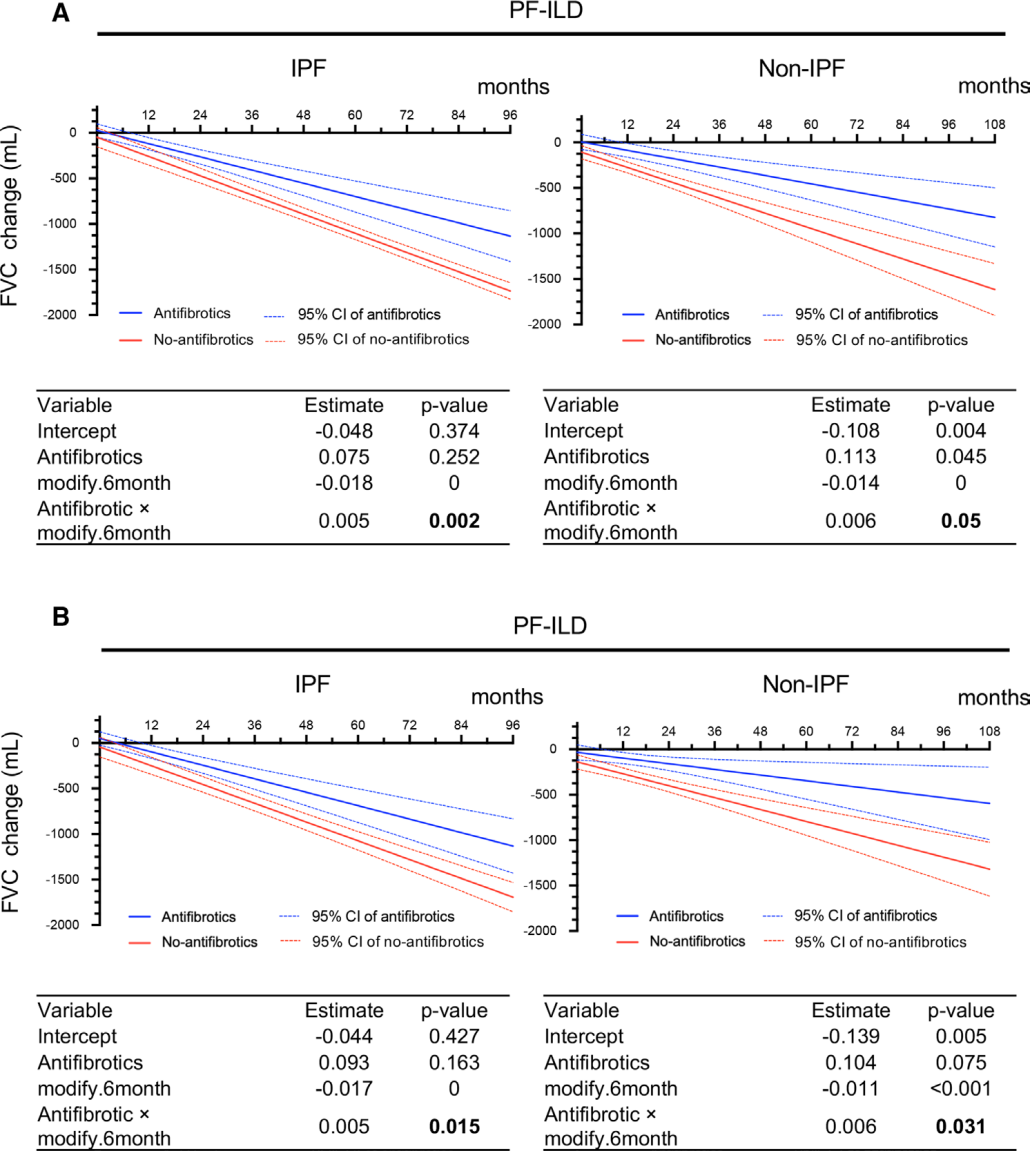
FVC change (mL) analysed by GEE at calculated time points in IPF and non-IPF. (A) Without adjustment. (B) With adjustment for multiple covariates^*^ using IPW. Each table shows a GEE analysis. Estimates and p values are shown for antifibrotics, month and the interaction between treatment and month. P values in bold indicate a significant effect. *Adjustment by age, sex, body mass index, FVC, glucocorticoid use (PSL at ≥10 mg/day), differential diagnoses (IPF, autoimmune ILD and lung-dominant ILD), and high-resolution CT findings (honeycombing and traction bronchiectasis). FVC, forced vital capacity; GEE, generalised estimating equation; IPF, idiopathic pulmonary fibrosis; IPW, inverse probability weighting; PF-ILD, progressive fibrosing interstitial lung disease; PSL, prednisolone.

In the antifibrotic (n=42) and no-antifibrotic (n=19) groups of patients with IPF, the unadjusted results from the GEE model on raw measures showed that the FVC decline from baseline was −45.32 and −153.27 mL at 6 months, −117.82 and −258.71 mL at 12 months, −552.77 and −891.33 mL at 48 months and −1132.71 and −1734.84 mL at 96 months of follow-up. In the antifibrotic (n=34) and no-antifibrotic (n=60) groups of patients with non-IPF, the unadjusted results from the GEE model on raw measures showed that the FVC decline from baseline was −41.21 and −191.04 mL at 6 months, −87.22 and −275.63 mL at 12 months, −363.25 and −777.80 mL at 48 months and −731.30 and −1447.37 mL at 96 months of follow-up (figure 2A).

The adjusted results obtained by IPW were similar. In the antifibrotic and no-antifibrotic groups of patients with IPF, the FVC decline from baseline was −24.33 and −147.07 mL at 6 months, −117.82 and −250.12 mL at 12 months, −245.73 and −456.21 mL at 24 months, −540.93 and −868.41 mL at 48 months and −1131.34 and −1692.8 mL at 96 months of follow-up. In the antifibrotic and no-antifibrotic groups of patients with non-IPF, the adjusted results obtained by IPW showed that the FVC decline from baseline was −65.87 and −204.55 mL at 6 months, −97.07 and −270.10 mL at 12 months, −159.48 and −401.22 mL at 24 months, −284.28 and −663.45 mL at 48 months, −409.09 and −925.67 mL at 72 months and −533.90 and −1187.90 mL at 96 months of follow-up (figure 2B).

In the no-antifibrotic group, patients with CTD-ILD had a smaller annual %FVC decline than patients with IIPs other than IPF, especially in the latter phase (p=0.0341) (online supplemental figure S3A, B).

10.1136/rmdopen-2022-002667.supp4 Supplementary data

In summary, the unadjusted and adjusted results of our analysis indicate that the antifibrotic group had a significantly smaller FVC decline than the no-antifibrotic group in both patients with IPF and those with non-IPF PF-ILD (p=0.002 and p=0.015 in IPF, p=0.05 and p=0.031 in non-IPF).

### OS

The mean follow-up time from PF-ILD diagnosis was 3.2±1.8 and 3.1±1.9 years in patients with IPF and non-IPF, respectively.

At the last follow-up, 22/64 (34.4%) patients with IPF and 30/103 (29.1%) patients with non-IPF had died: 35 deaths were due to respiratory failure, 4 were due to lung cancer, 5 were due to non-respiratory causes and 8 were due to unknown causes. There was no significant difference in the cause of death between the two disease groups, and the most frequent cause was associated with respiratory failure (online supplemental table S7).

**Figure 3:**
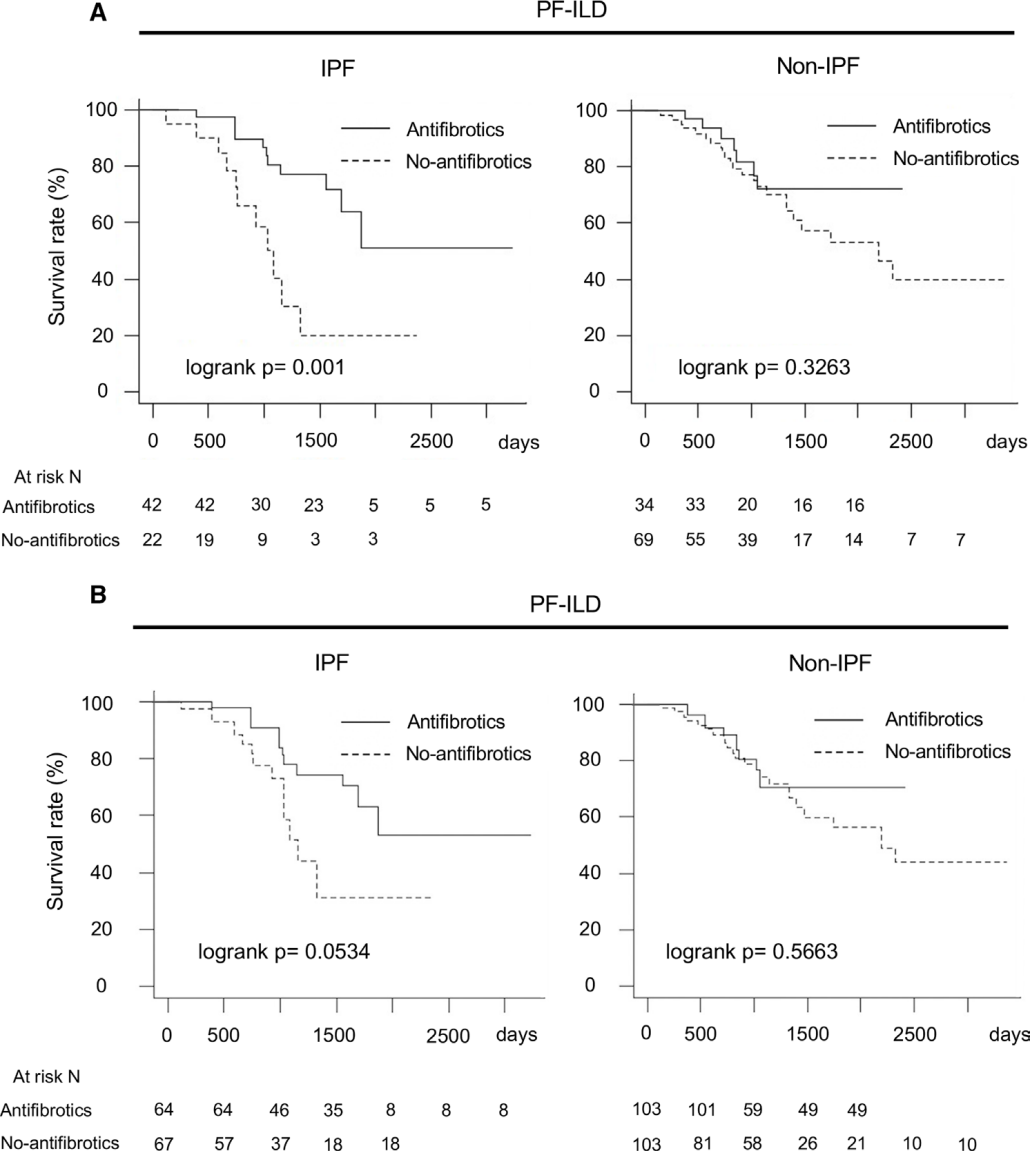
Comparison of overall survival in antifibrotic group and no-antifibrotic group in IPF and non-IPF. (A) Without adjustment. (B) With adjustment for multiple covariates^*^ using IPW. *Adjustment by age, sex, body mass index, FVC, glucocorticoid use (PSL at ≥10 mg/day), differential diagnoses (IPF, autoimmune ILD and lung-dominant ILD) and high-resolution CT findings (honeycombing and traction bronchiectasis). FVC, forced vital capacity; IPF, idiopathic pulmonary fibrosis; IPW, inverse probability weighting; PF-ILD, progressive fibrosing interstitial lung disease; PSL, prednisolone.

Among patients with IPF, OS was longer in those who did than did not receive antifibrotics (log-rank p=0.001) (figure 3A). Similar results were observed with IPW adjustment (log-rank p=0.0534) (figure 3B). In the antifibrotic and no-antifibrotic groups of patients with IPF, the adjusted OS rate was 97.6% (95% CI, 93.1% to 100%) and 95.2% (95% CI, 86.6% to 100%) at 12 months, 95.0% (95% CI, 88.4% to 100%) and 78.5% (95% CI, 61.8% to 99.8%) at 24 months and 78.2% (95% CI, 64.1% to 95.6%) and 51.5% (95% CI, 27.2% to 97.5%) at 36 months, respectively. In the antifibrotic and no-antifibrotic groups, the median survival times with adjustments were not reached and 36.2 months, respectively.

However, there was no such difference among patients with non-IPF (log-rank p=0.3263) (figure 3A). Similar results were observed with IPW adjustment (log-rank p=0.5663) (figure 3B).

In the antifibrotic and no-antifibrotic groups of patients with non-IPF, the adjusted OS rate was 96.4% (95% CI, 89.8% to 100%) and 94.2% (95% CI, 88.7% to 100%) at 12 months, 89.1% (95% CI, 78.0% to 100%) and 87.6% (95% CI, 79.8% to 96.2%) at 24 months and 70.7% (95% CI, 53.9% to 92.8%) and 74.1% (95% CI, 63.2% to 86.9%) at 36 months, respectively. In the antifibrotic and no-antifibrotic groups, the median survival times were not reached and 57.7 months, respectively.

**Figure 4:**
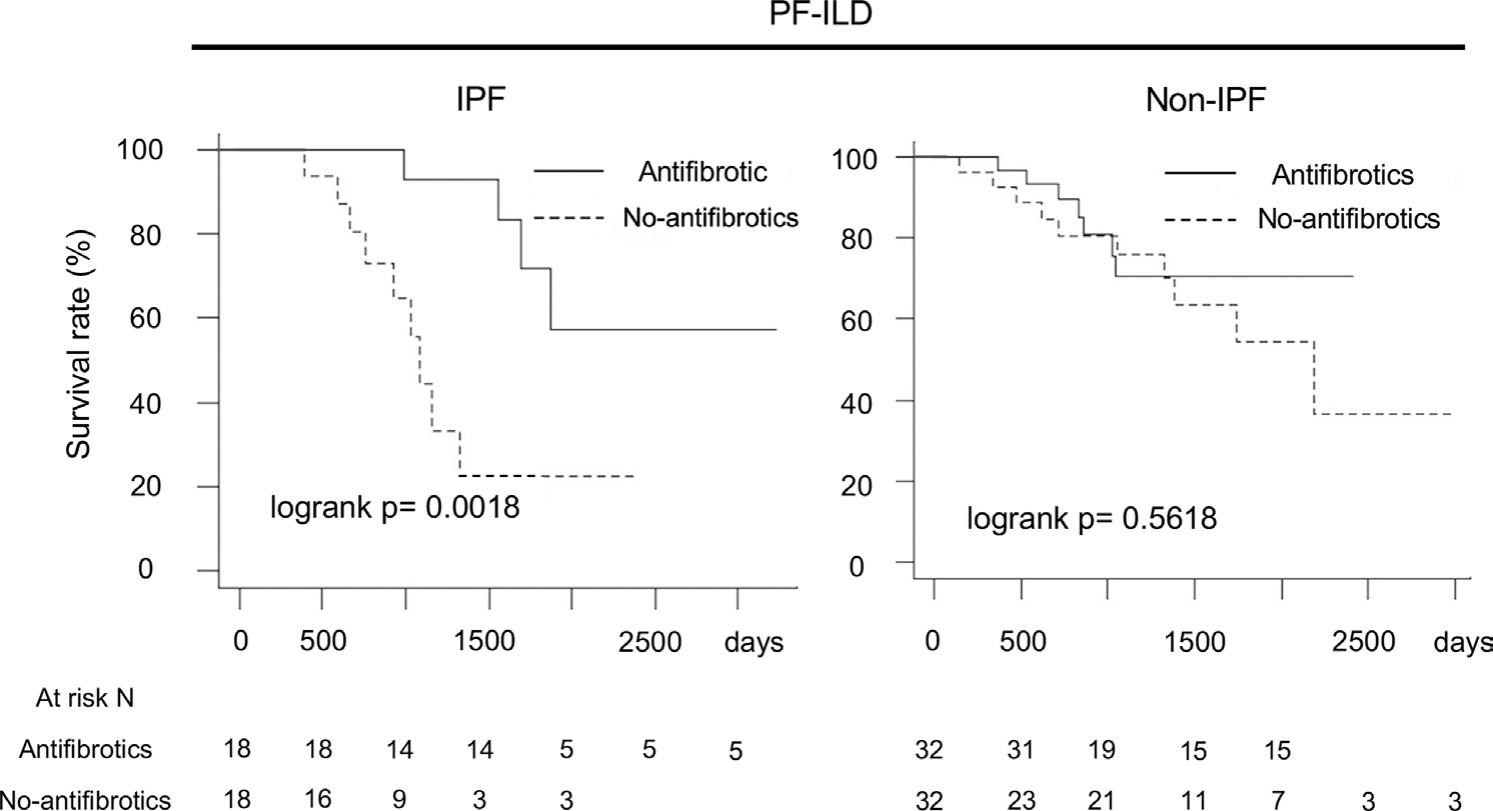
Comparison of overall survival in antifibrotic group and no-antifibrotic group using propensity score matching for adjustment of multiple covariates*. *Adjustment by age, sex, body mass index, FVC, glucocorticoid use (PSL at ≥10 mg/day), differential diagnoses (IPF, autoimmune ILD and lung-dominant ILD) and high-resolution CT findings (honeycombing and traction bronchiectasis). FVC, forced vital capacity; IPF, idiopathic pulmonary fibrosis; PF-ILD, progressive fibrosing interstitial lung disease; PSL, prednisolone.

For further performance of unbiased comparisons between the antifibrotic and no-antifibrotic groups, we assessed patient outcomes after 1:1 statistical matching using propensity scores. The propensity score-matched cohort analysis with the same preconfigured covariates showed the same results (IPF, log-rank p=0.0018; non-IPF, log-rank p=0.5618) (figure 4) (table 3 shows the baseline characteristics of patients in the IPF and non-IPF groups).

**Table 3:** Baseline characteristics of propensity score-matched patients with PF-ILD at PF-ILD diagnosis

	PF-ILD							
	IPF				Non-IPF			
	Matched total (n=36)	Matched antifibrotic (n=18)	Matched no-antifibrotic (n=18)	P value	Matched total (n=64)	Matched antifibrotic (n=32)	Matched no-antifibrotic (n=32)	P value
Characteristics								
Sex, male	30 (83.33)*	3 (16.67)	3 (16.67)	1.000	44 (68.75)	21 (65.63)*	23 (71.88)	0.7879
Age (years)	72.5 (69–75.75)	71 (67.75–76)	74 (69.75–78.5)	0.3939	71 (64–74)	71 (61.25–74)	71 (64–73)	0.7165
BMI	23.27 (21.40–25.15)	22.31 (19.60–25.22)	23.79 (21.91–25.10)	0.2503	22.79 (21.19–25.41)	22.70 (20.84–28.39)	23.76 (21.71–25.48)	0.6371
Serological examination								
Monocyte count	443.4 (352.2–578)	525.9 (375.6–604.4)	439.1 (339.3–551.4)	0.2466	435.4 (349.1–570.4)	435.4 (380.6–553.4)	436.5 (307.9–604.5)	0.7890
LDH	211 (184.5–265)	210 (184.3–266.3)	217 (188.5–265)	0.8148	215 (184.5–245.8)	211 (175.8–239.5)	223 (1882.5–255.5)	0.3901
KL-6	1087 (684.3–1644.8)	992 (696.3–1690.4)	1120.7 (614.5–1616.4)	0.9139	840.5 (592.7–1311.7)	805.5 (598.9–1311.7)	924.5 (454.6–1440.3)	0.8658
CRP	0.22 (0.1–0.49)	0.23 (0.1–0.53)	0.16 (0.1–0.46)	0.9620	0.15 (0.1–0.35)	0.17 (0.1–0.35)	0.13 (0.05–0.35)	0.3460
Pulmonary function								
FVC	2.34 (1.94–2.95)	2.34 (2.02–2.97)	2.48 (1.88–2.96)	0.9725	2.26 (1.73–2.92)	2.27 (1.84–2.78)	2.25 (1.73–3.07)	0.6182
%FVC	83.8 (71.5–91.33)	84.85 (72.78–90.93)	83.8 (70.33–91.75)	0.8207	79.1 (64.2–90.4)	79.3 (62.2–90.4)	78.5 (65.6–89.43)	0.6028
DLCO (n=74)	8.73 (7.18–11.08)	10.42 (7.7–11.19)	8.13 (7.07–10.36)	0.5093	9.13 (7.37–11.68)	9.05 (7.32–11.71)	10.07 (7.35–11.85)	0.7696
%DLCO (n=74)	56.6 (45.88–76.9)	62.26 (48.45–77.81)	55.6 (45.45–75.74)	0.5184	56.8 (42.4–71.75)	51.64 (40.48–72.65)	60.9 (43.5–71.6)	0.7720
Radiological findings								
Honeycombing	24 (66.67)	13 (72.22)	11 (61.11)	0.7247	19 (29.69)	10 (31.25)	9 (28.13)	1.000
Traction bronchiectasis	20 (55.56)	9 (50.0)	11 (61.11)	0.7380	44 (68.75)	21 (65.63)	23 (71.88)	0.7879
Immunosuppressive agents								
Glucocorticoids (PSL<10 mg/day)	0 (0.0)	0 (0.0)	0 (0.0)	1.000	5 (6.80)	3 (9.38)	2 (6.25)	1.000
Glucocorticoids (PSL≥10 mg/day)	7 (19.44)	4 (22.22)	3 (16.67)	1.000	10 (15.63)	4 (12.5)	6 (18.75)	0.7323
Immunosuppressant	2 (5.56)	1 (5.56)	1 (5.56)	1.000	11 (17.19)	6 (18.75)	5 (15.63)	1.000

*Data are shown as n (%) or median (IQR).

BMI, body mass index; CRP, C-reactive protein; DLCO, diffusing capacity for carbon monoxide; FVC, forced vital capacity; IPF, idiopathic pulmonary fibrosis; KL-6, sialylated carbohydrate antigen Krebs von den Lungen-6; LDH, lactate dehydrogenase; PF-ILD, progressive fibrosing interstitial lung disease; PSL, prednisolone.

Finally, we performed the same analysis including only the patients who underwent PFTs after the PF-ILD diagnosis, and the results were consistent with those described above (online supplemental figure S4A, B).

10.1136/rmdopen-2022-002667.supp5 Supplementary data

In summary, OS was longer in the antifibrotic group than in the no-antifibrotic group among patients with IPF. However, OS was not significantly different between the two groups among patients with non-IPF.

## Discussion

In this multicentre retrospective cohort study, we compared the FVC decline and prognosis in real-world PF-ILD between patients who did and did not receive antifibrotic agents using GEEs and IPW with adjustment for preconfigured covariates. This process enabled us to analyse repeated measures with the missing data from a single patient over time, statistically correct the heterogeneity of patient backgrounds and balance potential confounding factors. We showed the real-world efficacy of antifibrotics in patients with PF-ILD over a long-term follow-up. Regarding the prognosis, OS was longer in the antifibrotic group among patients with IPF. However, this finding was not observed among patients with non-IPF.

In patients with IPF, antifibrotics have been shown to improve FVC decline^34^ and OS.^35 36^ Furthermore, real-world studies and systematic reviews have confirmed the efficacy of antifibrotics in patients with IPF.^37 38^ Therefore, the prognostic effect of antifibrotic agents has been well established with robust external validity.

In patients with non-IPF PF-ILD, FVC decline is also associated with poor outcomes.^39^ IPF and PF-ILD have been suggested to share common fibrotic cascades that cause irreversible damage and poor outcomes.^40^ During the past few years, the SENSCIS trial has shown that nintedanib reduces the rate of FVC decline in patients with systemic sclerosis-associated ILD.^41^ Additionally, the INBUILD trial showed that nintedanib improved the FVC decline in patients with PF-ILD.^7^ Moreover, a recent subgroup analysis of the INBUILD trial showed improvement of the FVC decline regardless of the underlying ILD diagnosis,^8^ and a recent phase 2b trial showed a significantly lower decline in %FVC predicted in the pirfenidone than placebo group.^42^ These findings might suggest that antifibrotics improve the prognosis of non-IPF PF-ILD, similar to IPF.

However, in clinical trials, the enrolled patients receive strictly controlled treatment regimens with limited periods despite the fact that PF-ILDs encompass heterogeneous diseases and patient-specific variables.^43^ Indeed, because of the diversity of disease groups in PF-ILD, patients receive a wide variety of therapeutic interventions during the disease course.^25^

Therefore, the results may not be directly applicable to the broader patient population seen in clinical practice, and there is a lack of real-world data regarding whether antifibrotic agents really improve the FVC decline or prolong OS. Hence, we conducted the present study to reveal the efficacy of antifibrotics in real-world PF-ILD.

In this study, we convincingly showed that antifibrotic treatments significantly reduced FVC decline compared with no antifibrotic treatments in both patients with IPF and those with non-IPF PF-ILD. However, the prognostic impact of antifibrotic drug therapy differed between patients with IPF and non-IPF PF-ILD. OS was longer in the antifibrotic group than in the no-antifibrotic group among patients with IPF. However, OS was not significantly different in patients with non-IPF.

Hence, this study suggests that improving the FVC might not be synonymous with improving the prognosis of real-world non-IPF. Therefore, we should discuss why the results differed between IPF and non-IPF.

In clinical practice, immunosuppressive therapies are frequently administered for patients with non-IPF PF-ILD.^7 25 44^ Indeed, in the present study, patients with non-IPF frequently received immunosuppressive agents during the disease course of PF-ILD, especially GC use in the no-antifibrotic group (online supplemental table S2).

Many patients with IPF received GCs because of acute exacerbation of respiratory symptoms (17/22 (77.3%)). Among patients with CTD-ILD, which is characterised by not only pulmonary lesions but also systemic pathology, GCs are generally used for disease control.

In particular, GC therapy is the standard treatment for myopathy-associated ILD.^45^ Indeed, in the no-antifibrotic group, patients with CTD-ILD had a smaller annual %FVC decline than patients with IIPs other than IPF, especially in the latter phase (p=0.0341), which is consistent with the previous report (online supplemental figure S3A, B).^45^

Although the effect of immunosuppressive agents remains unclear in PF-ILD, we cannot deny that anti-inflammatory treatment for systemic disease control might change the disease course, preventing detection of the prognostic effect of antifibrotic drugs alone.

The lack of effects of antifibrotics on mortality may have also been driven by the low number of patients in the non-IPF group. Because the survival rate was generally much higher in the patients with non-IPF in this study, a longer follow-up time and much higher numbers are likely needed to detect differences in survival with any intervention for non-IPF. We calculated the sample sizes needed for detection of a survival difference based on our cohort data. A larger sample size of >228 with a longer follow-up (48 months) might detect differences from the present study (the required sample size was estimated based on median survival time of 57.7 months in the no-antifibrotic group, HR of 58.1% (estimated using a Cox proportional hazard model), 1:1 allocation, 80% power and alpha value of 0.05 (two-sided) using the binomial test).^46^ Because our results showed the prognostic effect of antifibrotic agents in patients with IPF, consistent with previous reports with robustness, the results showed the validity of the cohort itself.

In summary, we need to investigate the prognostic effects of antifibrotics on non-IPF PF-ILD using a larger cohort with a longer observation time, ideally in consideration of fluctuations in the intensity of anti-inflammatory immunosuppressive therapy during treatment. Our results and discussion will provide insight into further understanding of PF-ILD.

This study had several important limitations. First, we could not exclude potential confounding factors because of the retrospective nature of the study. Several unknown confounders may be present in newly defined progressive phenotypes. Although not statistically significant, DLCO and %DLCO were smaller in the no-antifibrotic group than in the antifibrotic group of patients with IPF (table 2 and online supplemental table S6), which could have served as a confounding factor. However, there was a considerable number of missing values (DLCO and %DLCO, n=19/64; FVC and %FVC, n=0). Furthermore, adjusting them as covariates would lead to lower statistical power of our study because missing cases needed to be excluded. Hence, we incorporated general covariates from previous reports (well discussed, especially in IPF) with validity.^22–29 35 38^

Second, because of the retrospective study design, the attending pulmonary physicians made decisions independently regarding the timing and selection of antifibrotic agents. Although a previous report showed the effect of pirfenidone in PF-ILD,^47^ the guideline recommends further research into the efficacy of pirfenidone.^2^ Some patients with non-IPF receive pirfenidone (online supplemental table S8 shows the prescription between nintedanib and pirfenidone in each group). We compared the effects of pirfenidone and nintedanib and found no significant difference (mean %FVC change/year, p=0.3212; OS, log-rank p=0.3003) (online supplemental figure S5A, B).

10.1136/rmdopen-2022-002667.supp6 Supplementary data

We also considered the reasons why 22 patients with IPF did not receive antifibrotics despite well-established evidence. Reports from Europe have shown that conventional treatments (steroids/immunosuppressants) have been eliminated and replaced by antifibrotic drugs.^38^ However, despite the availability of antifibrotics in Japan, Tomioka *et al*^48^ reported that many patients with IPF in Japan still do not receive antifibrotics. As shown in online supplemental table S1, six patients did not request antifibrotics because of concerns regarding side effects. We were unable to confirm whether options for antifibrotic agents were presented to about another six patients. One of the reasons is that the study period started several years before the establishment of survival benefits of antifibrotics, and the physicians’ understanding of antifibrotic agents and the informed consent obtained from elderly patients and patients with few subjective symptoms might have been insufficient. Another reason was a financial burden in two patients.

Third, our study population included all patients who met the diagnostic criteria for PF-ILD. Twelve patients were censored from the analysis of how antifibrotics affect lung function because of missing PFT results after the PF-ILD diagnosis. Hence, the lung function evaluation may have been affected by selection bias. However, the exclusion of these patients did not distort the patient backgrounds (table 2 and online supplemental table S6). Furthermore, we performed survival analyses of the entire study population as well as the study population after excluding the patients lacking PFTs after the PF-ILD diagnosis and obtained the same results (online supplemental figure S4A, B). Therefore, our cohorts were valid and showed consistency.

Fourth, because patients visit medical institutions at various times in the real-world setting, our study may not reflect a completely accurate time period and FVC decline for patient inclusion despite the strict rule with window periods of PFTs. Therefore, whether the study population reflects true progressive phenotypes is unclear, and the results may have been affected by selection bias. However, FVC changes and OS were different between the non-PF-ILD and PF-ILD groups (online supplemental figure S1A, B and S2). Hence, we consider that the patients with PF-ILD in our study had a distinct progressive phenotype.

10.1136/rmdopen-2022-002667.supp2 Supplementary data

Fifth, the endpoint of our study was death or the end of follow-up. This could have affected the interpretation of our results because of measurement bias. However, we assessed the patients for as long as possible through the regional medical liaison office. Moreover, even if a patient had been followed up at another hospital, we received subsequent medical information as specialised hospitals; thus, there was little loss to death.

Finally, this study was conducted in referral centres in Japan. Applicability to a broader population should be examined in future studies, especially external to Asia.

Although the total number of deaths was higher in this study (IPF, 22/64 (34.4%); non-IPF, 30/103 (29.1%)) than in previous studies, which may have been associated with the fact that the median age was more than 5 years older than the study from Europe,^49^ the rate of respiratory failure in patients with non-IPF was similar to that in a previous report (20/30 (66.7%)).^25^ Our study population reflects the regional real-world epidemiology in Japan. Accumulation of additional data from future research will reveal the worldwide epidemiology of PF-ILD.

In conclusion, this is the first real-world study to show that antifibrotics improve the FVC decline in patients with PF-ILD. However, among patients with non-IPF, the mortality rate was not significantly different between those with and without antifibrotics, suggesting the need for further studies.

10.1136/rmdopen-2022-002667.supp3 Supplementary data

## Data Availability

All data relevant to the study are included in the article or uploaded as supplementary information.
