# Supplementary material for: Real-world impact of antifibrotics on prognosis in patients with progressive fibrosing interstitial lung disease
Source: RMD Open. 2023 Jan 23;9(1):e002667. doi: 10.1136/rmdopen-2022-002667 (PMC9872509; doi:10.1136/rmdopen-2022-002667)
Supplement: Supplementary data [file rmdopen-2022-002667supp001.pdf]

Supplemental Table S1.

Cause	IPF* (n=22)
Discontinuation for adverse event or emerging complications	4
Cardiac disease	1
Kidney injury	1
Rash	1
Lung injury	1
Patient's request owing to concerns regarding side effects	6
Social problem[1]	2
The medical judgment of the attending physician[2]	4
Others[3]	6

Supplemental Table S1. The reason for no antifibrotic therapy in IPF patients during the study period.

[1] Financial burden.

[2] Considered elderly.

[3] Unknown or unable to confirm whether options for antifibrotic agents were presented.

*Abbreviations: IPF, idiopathic pulmonary fibrosis.

Supplemental Table S2.

	PF-ILD*							
	IPF				Non-IPF			
	Total (n=64)	Antifibrotic (n=42)	Without antifibrotic (n=22)	P value§	Total (n=103)	Antifibrotic (n=34)	Without antifibrotic (n=69)	P value
Glucocorticoids	22 (34.38)	10 (23.81)	12 (54.55)	0.0139	61 (59.22)	16 (47.06)	45 (65.22)	0.0911
Immunosuppressant	5 (7.81)	1 (2.38)	4 (18.18)	0.0437	22 (21.36)	7 (20.59)	15 (21.74)	1.000

Supplemental Table S2. Immunosuppressive agents received during the study period in IPF and non-IPF.

‡Data are shown as n (%).

§Differences were assessed using the $\chi 2$ test or Fisher's exact test, as appropriate.

*Abbreviations: PF-ILD, progressive fibrosing interstitial lung disease; IPF, idiopathic pulmonary fibrosis.

Supplemental Table S3.

	PF-ILD*							
			Non-IPF (n=103)					
	Total (n=167)	IPF (n=64)	IIPs other than IPF (n=49) NSIP (n=19), PPFE (n=2) Unclassified (n=26), COP (n=2)	CTD-ILD (n=31) SSc (n=12), RA (n=8), IM[1] (n=7), Others[2] (n=4)	IPAF (n=7)	HP (n=10)	Others[3] (n=6)	P value§
Glucocorticoids	83 (49.7)	22 (34.38)	26 (53.06)	24 (77.42)	4 (57.14)	6 (60.0)	1 (16.66)	0.0011
Immunosuppressant	27 (16.17)	5 (7.81)	7 (14.29)	14 (45.16)	1 (14.29)	0 (0.0)	0 (0.0)	<0.0001

Supplemental Table S3. Immunosuppressive agents received during the study period in each disease groups.

[1]Dermatomyositis (n=4), Polymyositis (n=3)

[2]Microscopic Polyangiitis (n=2), Mixed Connective Tissue Disease (n=1), Systemic lupus erythematosus (n=1).

[3]Exposure-related ILD (n=5), Sarcoidosis (n=1)

‡Data are shown as n (%).

§Differences were assessed using the $\chi 2$ test or Fisher's exact test, as appropriate.

Abbreviations: PF-ILD, progressive fibrosing interstitial lung disease; IPF, idiopathic pulmonary fibrosis; IIPs, idiopathic interstitial pneumonias; NSIP, non-specific interstitial pneumonia; PPFE, pleuroparenchymal fibroelastosis; COP, cryptogenic organizing pneumonia; CTD-ILD, connective tissue disease-interstitial lung disease; SSc, systemic sclerosis; IM, inflammatory myopathies; RA, rheumatoid arthritis; IPAF, interstitial pneumonia with autoimmune features; HP, hypersensitivity pneumonitis.

Supplemental Table S4.

Immunosuppressant	IPF* (n=5)	Non-IPF (n=22)
Cyclosporine A	5	12
Mycophenolate mofetil	0	9
Cyclophosphamide	1	3
Tacrolimus	0	3
Azathioprine	0	2
Tocilizumab	0	3

	Glucocorticoid	Cyclosporine A	Mycophenolate mofetil	Cyclophosphamide	Tacrolimus	Azathioprine	Tocilizumab
Systemic sclerosis (n=12)	10	1	7	2	0	1	3
Rheumatoid arthritis (n=8)	5	2	0	0	0	0	0
Dermatomyositis (n=4)	2	0	0	0	0	0	0
Polymyositis (n=3)	3	1	0	0	1	0	0
Microscopic Polyangiitis (n=2)	2	0	0	1	0	0	0
Mixed Connective Tissue Disease (n=1)	1	0	0	0	0	0	0
Systemic lupus erythematosus (n=1)	1	1	0	0	0	0	0

Supplemental Table S4. The details of immunosuppressant received during the study period in in IPF and non-IPF, CTD-ILDs.

*Abbreviations: IPF, idiopathic pulmonary fibrosis; CTD-ILD, connective tissue disease-interstitial lung disease.

Supplemental Table S5.

	Excluded		PF-ILD*	
	Non-PF-ILD (n=383)	Low %FVC (<45%) (n=20)		
	IPF (n=46), IIps other than IPF (n=137), CTD-ILD (n=144), HP (n=9), IPAF (n=27), others (n=20)	IPF (n=1), IIps other than IPF (n=7), CTD-ILD (n=7), HP (n=1), IPAF (n=3), others (n=1)	Total (n=167)	P value§
**Characteristics**				
Sex, male	205 (53.51) ‡	11 (55.0)	115 (68.86)	0.0035
Age (years)	68 (61.0-74.0)	52 (43.25-65.25)	68.5 (62.5-72.0)	<0.0001
BMI	22.76 (20.89-24.85)	23.20 (19.46-25.83)	22.84 (21.22-25.33)	0.8181
COPD	58 (15.85)	0 (0.0)	20 (13.07)	0.1385
DM	90 (24.66)	3 (16.67)	24 (14.37)	0.1112
CTD-ILD	144 (37.6)	7 (35.00)	31 (18.56)	<0.001
**Serological examination**				
Monocyte count	401.3 (300.-520.6)	456.5 (329.4-571.4)	400.8 (319.5-508.3)	0.6033
LDH	210 (183-245.8)	253 (211.5-336.8)	219 (192-246.5)	0.0078
KL-6	658 (389.8-1126.3)	1115 (791.18-3363.3)	922 (510-1310)	<0.0001
CRP	0.04 (0.1-0.32)	0.33 (0.11-0.44)	0.13 (0.05-0.35)	0.1690
**Pulmonary function**				
FVC	2.6 (1.97-3.22)	1.36 (1.05-1.52)	2.61 (2.08-3.14)	<0.0001
%FVC	86.4 (71.9-92.9)	38.75 (34.48-42.55)	83.3 (71.9-92.9)	<0.0001
DLCO (n=402)	10.93 (8.05-14.74)	3.95 (1.73-6.59)	10.07 (7.47-12.0)	<0.0001
%DLCO (n=402)	66.5 (48.2-82.1)	20.2 (10.35-35.75)	61.75 (47.8-76.32)	<0.0001
**Radiological findings**				
Honeycombing	70 (18.28)	4 (20.0)	65 (38.92)	<0.0001
Traction bronchiectasis	170 (44.39)	12 (60.0)	101 (60.48)	0.0010
**Immunosuppressive agents during the study period**				
Glucocorticoids	192 (50.13)	15 (75.0)	82 (49.1)	<0.0001
Immunosuppressant	115 (30.03)	12 (60.0)	28 (16.77)	<0.0001
**Outcome**				
Death	35 (9.49)	2 (10.53)	52 (31.14)	<0.001

Supplemental Table S5. Characteristics of excluded patients and PF-ILD patients at first visit.

‡Data are shown as n (%) or median (interquartile range).

§Differences among continuous variables were assessed using the Kruskal-Wallis test; differences among categorical variables were assessed using the χ^2 test or Fisher's exact test, as appropriate. The Steel-Dwass test for multiple comparisons was used for statistical analyses of three or more groups.

*Abbreviations: PF-ILD, progressive fibrosing interstitial lung disease; IPF, idiopathic pulmonary fibrosis; IIPs, idiopathic interstitial pneumonias; CTD-ILD, connective tissue disease-interstitial lung disease; IPAF, interstitial pneumonia with autoimmune features; HP, hypersensitivity pneumonitis; BMI, body mass index; COPD; chronic obstructive lung pulmonary disease; DM, diabetes mellitus; LDH, lactate dehydrogenase; KL-6, sialylated carbohydrate antigen Krebs von den Lungen-6; CRP, C-reactive protein; FVC, forced vital capacity; DLCO, diffusing capacity for carbon monoxide.

Supplemental Table S6.

	Excluded	PF-ILD*								
	Lacking pulmonary function test after PF-ILD diagnosis (n=12)	Total (n=155)	IPF				Non-IPF			
			Total (n=61)	Antifibrotic (n=42)	No-antifibrotic (n=19)	P value§	Total (n=94)	Antifibrotic (n=34)	No-antifibrotic (n=60)	P value
**Characteristics**										
Sex, male	10 (83.33)‡	105 (67.74)	45 (73.77)	29 (69.05)	16 (84.21)	0.3464	60 (63.83)	24 (70.59)	36 (60.0)	0.5007
Age (years)	71.0 (62.5-74.25)	71.0 (64.0-75.0)	73.0 (68.5-77.0)	71.5 (66.75-76.25)	74 (71.0-78.0)	0.1467	69 (60.8-74.0)	69.5 (63.25-74)	69 (62.5-74.0)	0.6086
BMI	22.36 (21.70-24.81)	22.83 (21.13-25.39)	23.73 (21.77-26.31)	23.81 (21.63-26.71)	23.36 (21.83-25.39)	0.8274	22.45 (20.21-25.07)	23.57 (20.98-26.47)	21.91 (19.86-24.26)	0.0156
COPD	3 (25.00)	21 (13.54)	7 (14.06)	5 (11.9)	2 (10.53)	0.8816	14 (12.62)	7 (20.59)	7 (11.48)	0.2341
DM	0 (0.00)	24 (15.48)	11 (17.19)	5 (11.9)	6 (31.58)	0.1216	13 (12.62)	3 (8.82)	10 (14.49)	0.7168
**Serological examination**										
Monocyte count	336 (286.4-693.4)	432.9 (337.9-539)	420.5 (333.3-527.1)	402.6 (329.67-539)	439.1 (339.8-498.4)	0.8292	433.2 (340.7-552)	437.7 (379.8-554.7)	429.2 (302.1-551.5)	0.3584
LDH	218.5 (161.5-259.3)	217 (194.5-250.3)	216 (193.5-260.0)	214 (192-248)	217 (196.5-274)	0.5564	217.5 (193.8-245.5)	213 (181.5-246.5)	224.5 (200.5-250.3)	0.3037
KL-6	647.5 (403.2-1496.8)	898.5 (590.7-1357.2)	948.3 (700.4-1554.8)	916.4 (740.5-1606)	1058.9 (614.5-1510.6)	0.9645	852 (564-1269)	829 (598.6-1423.1)	871 (464.1-1166.6)	0.2519
CRP	0.30 (0.1-1.28)	0.16 (0.1-0.36)	0.16 (0.1-0.43)	0.16 (0.1-0.32)	0.19 (0.11-0.46)	0.4010	0.15 (0.1-0.35)	0.18 (0.1-0.35)	0.14 (0.07-0.35)	0.5074
**Pulmonary function**										
FVC	2.13 (1.72-2.57)	2.31 (1.81-2.85)	2.29 (1.91-2.84)	2.29 (1.89-2.87)	2.35 (1.91-2.83)	0.9438	2.33 (1.79-2.89)	2.33 (1.96-2.77)	2.41 (1.75-3.07)	0.6193
%FVC	84.15 (72.7-92.78)	82.1 (68.0-91.6)	84.3 (71.8-93.15)	86.55 (72.83-94.0)	84.1 (68.9-91.7)	0.2916	78.8 (65.1-90.4)	79.3 (63.0-90.75)	79.2 (65.6-89.6)	0.5710
DLCO (n=125)	9.51 (3.64-15.26)	10.07 (7.47-12.0)	9.96 (7.88-12.0)	11.04 (8.41-12.74)	8.47 (7.07-10.36)	0.0226	10.07 (7.33-11.98)	9.69 (7.26-11.76)	10.29 (7.49-12.64)	0.6082
%DLCO (n=125)	64.26 (20.20-79.85)	58.5 (44.6-75.1)	59.17 (46.65-77.44)	69.66 (49.05-80.97)	47.8 (41.4-57.77)	0.0280	58.5 (42.95-72.8)	52.8 (40.03-71.75)	61.5 (43.73-74.68)	0.2074
**Radiological findings**										
Honeycombing	6 (50.0)	59 (38.06)	34 (55.74)	22 (52.38)	12 (63.16)	0.5792	25 (26.6)	12 (35.29)	13 (21.67)	0.1452
Traction bronchiectasis	5 (41.67)	96 (61.94)	39 (63.93)	27 (64.29)	12 (63.16)	1.000	57 (58.25)	22 (64.71)	35 (58.33)	0.6614
**Immunosuppressive agents**										
Glucocorticoids (PSL<10mg/day)	1 (8.33)	11 (7.1)	0 (0.0)	0 (0.0)	0 (0.0)	1.000	11 (11.70)	3 (8.82)	8 (13.33)	0.7414
Glucocorticoids (PSL≧10mg/day)	5 (41.67)	18 (11.67)	6 (9.84)	4 (9.52)	2 (10.53)	1.000	12 (12.77)	5 (14.71)	7 (11.67)	0.7515
Immunosuppressant	1 (8.33)	15 (9.68)	2 (3.28)	1 (2.38)	1 (5.36)	0.5295	13 (13.83)	6 (17.65)	7 (11.67)	0.5360

Supplemental Table S6. Baseline characteristics of patients with PF-ILD between the antifibrotic and no antifibrotic group at PF-ILD diagnosis and except the patients with lacking PFTs after PF-ILD diagnosis.

‡Data are shown as n (%) or median (interquartile range).

§Differences among continuous variables were assessed using Welch's t-test for monocyte count and pulmonary function tests (PFTs) and the Wilcoxon rank sum test for other variables; differences among categorical variables were assessed using the χ2 test or Fisher's exact test, as appropriate.

*Abbreviations: PF-ILD, progressive fibrosing interstitial lung disease; IPF, idiopathic pulmonary fibrosis; BMI, body mass index; COPD; chronic obstructive lung pulmonary disease; DM, diabetes mellitus; LDH, lactate dehydrogenase; KL-6, sialylated carbohydrate antigen Krebs von den Lungen-6; CRP, C-reactive protein; FVC, forced vital capacity; DLCO, diffusing capacity for carbon monoxide; PSL, prednisolone.

Supplemental Table S7.

	PF-ILD*							
			Non-IPF (n=103)					
	**Total (n=167)**	**IPF (n=64)**	IIPs other than IPF (n=49) NSIP (n=19), PPFE (n=2) Unclassified (n=26), COP (n=2)	CTD-ILD (n=31), SSc (n=12), RA (n=8), IM[1] (n=7), Others[2] (n=4)	IPAF (n=7)	HP (n=10)	Others[3] (n=6)	P value§
Total death	52 (31.14) ‡	22 (34.38)	17 (34.69)	8 (25.81)	2 (28.57)	3 (30.0)	0 (0.00)	0.6287
Respiratory failure[4]	35 (23.44)	15 (23.44)	12 (24.49)	4 (12.90)	1 (14.29)	3 (30.0)	0 (0.00)	0.6029
Lung cancer	4 (2.4)	1 (1.56)	2 (4.08)	0 (0.00)	1 (14.29)	0 (0.00)	0 (0.00)	0.2861
Others	5 (2.99)	1 (1.56)	1 (2.04)	3 (9.68)[5]	0 (0.00)	0 (0.00)	0 (0.00)	0.3871
Unknown	8 (4.79)	5 (7.81)	2 (3.92)	1 (3.23)	0 (0.00)	0 (0.00)	0 (0.00)	0.9157

Supplemental Table S7. The cause of death in each disease groups.

[1]Dermatomyositis (n=4), Polymyositis (n=3)

[2]Microscopic Polyangiitis (n=2), Mixed Connective Tissue Disease (n=1), Systemic lupus erythematosus (n=1).

[3]Exposure-related ILD (n=5), Sarcoidosis (n=1)

[4]Respiratory failure was defined as a disease progression, acute exacerbation, and complication of ILD other than lung cancer (e,g pneumothorax).

[5]Colon cancer (n=1), Heart failure (n=1), infection (n=1)

‡Data are shown as n (%).

§Differences among continuous variables were assessed using the one-way analysis of variance; differences among categorical variables were assessed using the $\chi 2$ test or Fisher's exact test, as appropriate.

*Abbreviations: IPF, idiopathic pulmonary fibrosis; IIPs, idiopathic interstitial pneumonias; NSIP, non-specific interstitial pneumonia; PPFE, pleuroparenchymal fibroelastosis; COP, cryptogenic organizing pneumonia; CTD-ILD, connective tissue disease-interstitial lung disease; SSc, systemic sclerosis; IM, inflammatory myopathies; RA, rheumatoid arthritis; IPAF, interstitial pneumonia with autoimmune features; HP, hypersensitivity pneumonitis.

Supplemental Table S8.

Antifibrotic agents	IPF* (n=42)	non-IPF (n=34)
Nintedanib	17	20
Pirfenidone	25	14

Supplemental Table S8. The prescription between nintedanib and pirfenidone in each group.

*Abbreviations: IPF, idiopathic pulmonary fibrosis.
