# Supplementary material for: Real-world impact of antifibrotics on prognosis in patients with progressive fibrosing interstitial lung disease
Source: RMD Open. 2023 Jan 23;9(1):e002667. doi: 10.1136/rmdopen-2022-002667 (PMC9872509; doi:10.1136/rmdopen-2022-002667)
Supplement: Supplementary data [file rmdopen-2022-002667supp004.pdf]

Supplemental Figure S3.

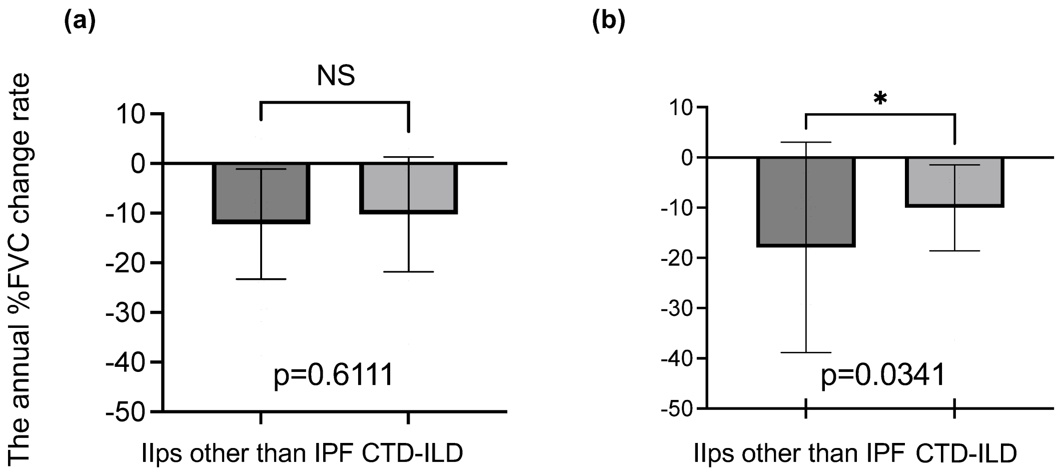

The annual %FVC change rate between IIps other than IPF and CTD-ILD in the no antifibrotic group for (a) the 1st year after PF-ILD diagnosis and (b) the observation period after PF-ILD diagnosis.

＊Differences were assessed using the Wilcoxon rank sum test.

Abbreviations: %FVC; percentage of forced vital capacity; NS, not significant; IIPs, idiopathic interstitial pneumonias; IPF, idiopathic pulmonary fibrosis; CTD-ILD, connective tissue disease-related interstitial lung disease.
