# Supplementary material for: Real-world impact of antifibrotics on prognosis in patients with progressive fibrosing interstitial lung disease
Source: RMD Open. 2023 Jan 23;9(1):e002667. doi: 10.1136/rmdopen-2022-002667 (PMC9872509; doi:10.1136/rmdopen-2022-002667)
Supplement: Supplementary data [file rmdopen-2022-002667supp005.pdf]

Supplemental Figure S4.

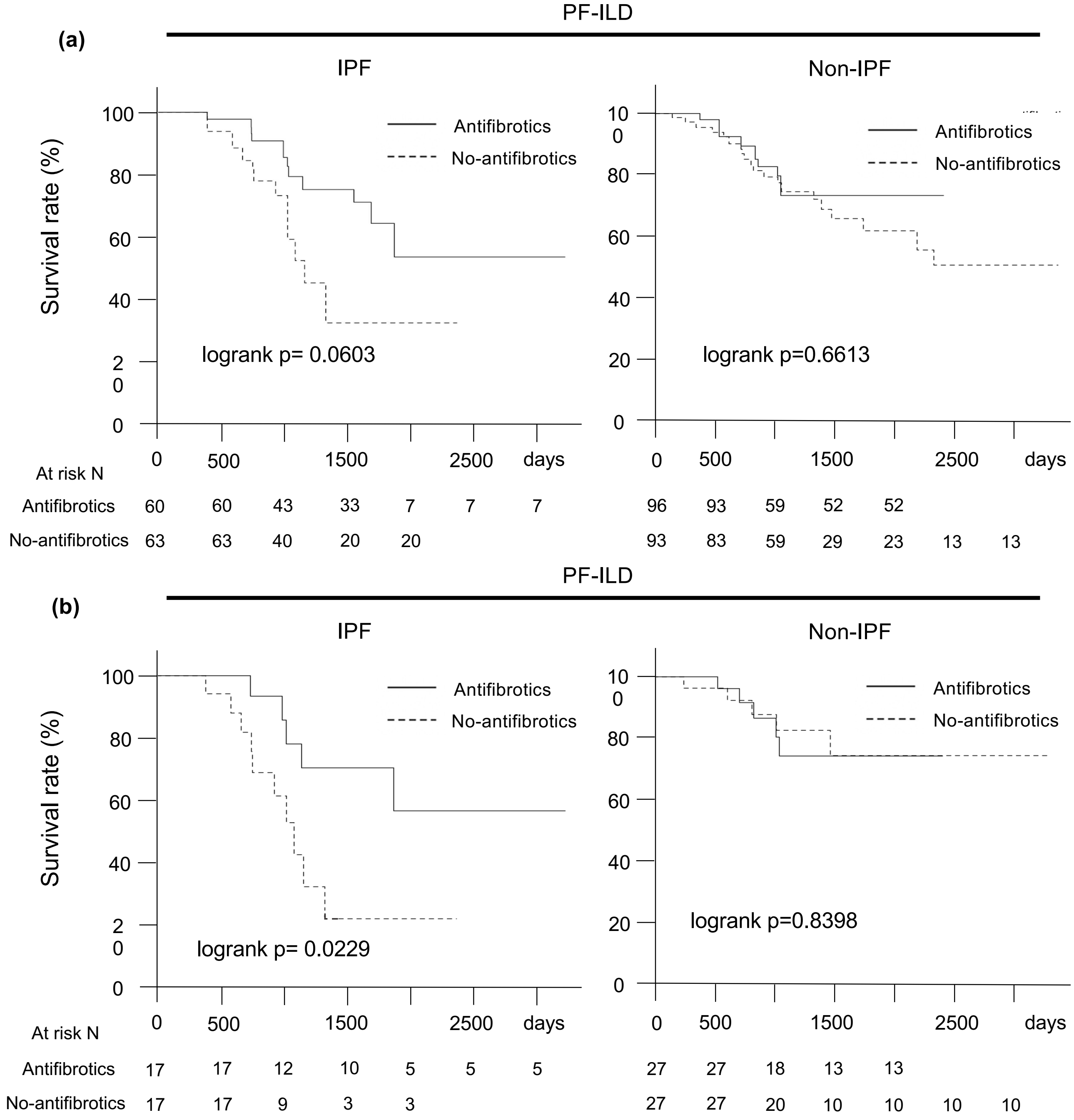

Survival estimates were performed using the Kaplan–Meier method and compared by log-rank test between antifibrotic group and no-antifibrotic group adjustment for multiple covariates＊ using
(a) IPW and (b) statistical matching in IPF and non-IPF.
＊Adjustment by age, sex, body mass index, FVC, glucocorticoid use (PSL at ≥10 mg/day), differential diagnoses (IPF, autoimmune ILD, and lung-dominant ILD), and high-resolution computed tomography findings (honeycombing and traction bronchiectasis)
Abbreviations: IPF, idiopathic pulmonary fibrosis; PF-ILD, progressive fibrosing interstitial lung disease; IPW, inverse probability weighting; FVC, forced Vital Capacity; PSL, prednisolone.
