# Supplementary material for: Real-world impact of antifibrotics on prognosis in patients with progressive fibrosing interstitial lung disease
Source: RMD Open. 2023 Jan 23;9(1):e002667. doi: 10.1136/rmdopen-2022-002667 (PMC9872509; doi:10.1136/rmdopen-2022-002667)
Supplement: Supplementary data [file rmdopen-2022-002667supp006.pdf]

Supplemental Figure S5.

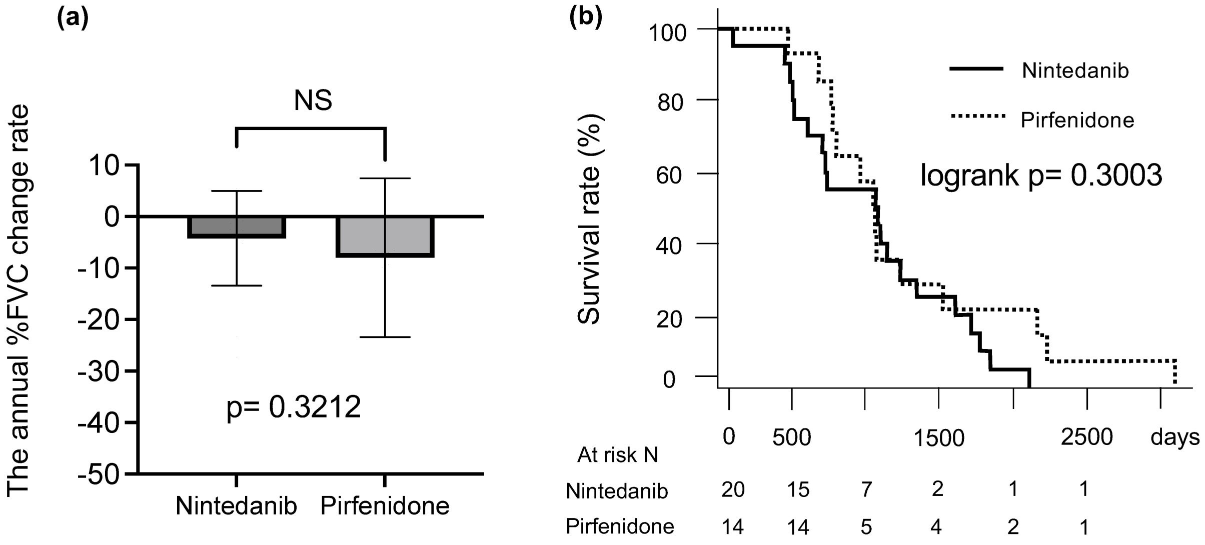

Comparison of the effects of the patients prescribed nintedanib (n=20) and those prescribed pirfenidone (n=14) in the non-IPF antifibrotic group.

(a) The annual %FVC change rate / year were compared by the Wilcoxon rank sum test.

(b) Survival estimates were performed using the Kaplan–Meier method and compared by log-rank test.

Abbreviations: %FVC; percentage of forced vital capacity; NS, not significant.
