# Supplementary material for: Real-world impact of antifibrotics on prognosis in patients with progressive fibrosing interstitial lung disease
Source: RMD Open. 2023 Jan 23;9(1):e002667. doi: 10.1136/rmdopen-2022-002667 (PMC9872509; doi:10.1136/rmdopen-2022-002667)
Supplement: Supplementary data [file rmdopen-2022-002667supp002.pdf]

Supplemental Figure S1.

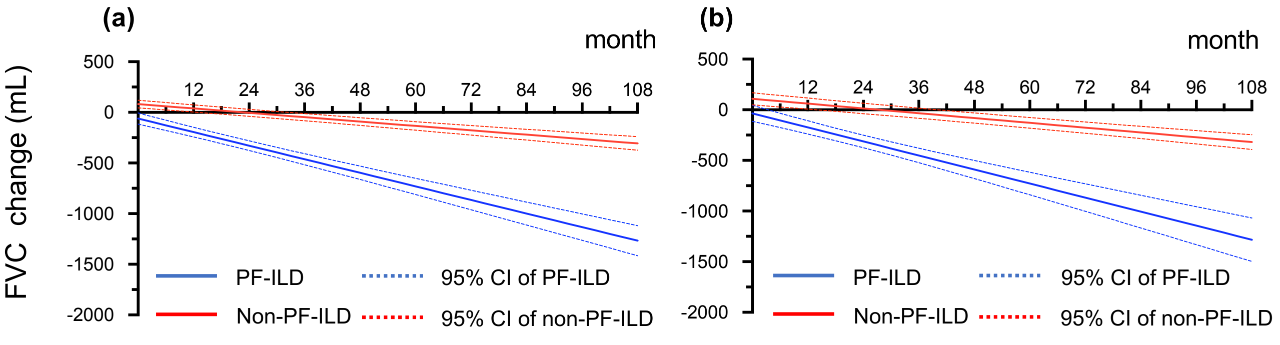

(a)

Variable	Estimate	p-value
Intercept	0.0819	<0.001
PF or non PF × modify.12month	-0.008	<0.001

(b)

Variable	Estimate	p-value
Intercept	0.108	<0.001
PF or non PF × modify.12month	-0.004	<0.001

The comparison of FVC between the PF-ILD and non-PF-ILD.
(a) unadjusted, and (b) adjusted change in FVC and groups was analyzed by GEE at calculated time points using IPW with adjustment for the multiple covariates＊ using propensity scores.
For this analysis, FVC measurements by year were attributed by the nearest measurement using a ±6 months window.
＊Adjustment by age, sex, body mass index, FVC, glucocorticoid use (PSL at ≥10 mg/day), differential diagnoses (IPF, autoimmune ILD, and lung-dominant ILD), and high-resolution computed tomography findings (honeycombing and traction bronchiectasis)
Abbreviations: FVC, forced vital capacity; PF-ILD, progressive fibrosing interstitial lung disease; GEE, generalized estimating equation; IPW, inverse probability weighting.
