# Supplementary material for: Real-world impact of antifibrotics on prognosis in patients with progressive fibrosing interstitial lung disease
Source: RMD Open. 2023 Jan 23;9(1):e002667. doi: 10.1136/rmdopen-2022-002667 (PMC9872509; doi:10.1136/rmdopen-2022-002667)
Supplement: Supplementary data [file rmdopen-2022-002667supp003.pdf]

Supplemental Figure S2.

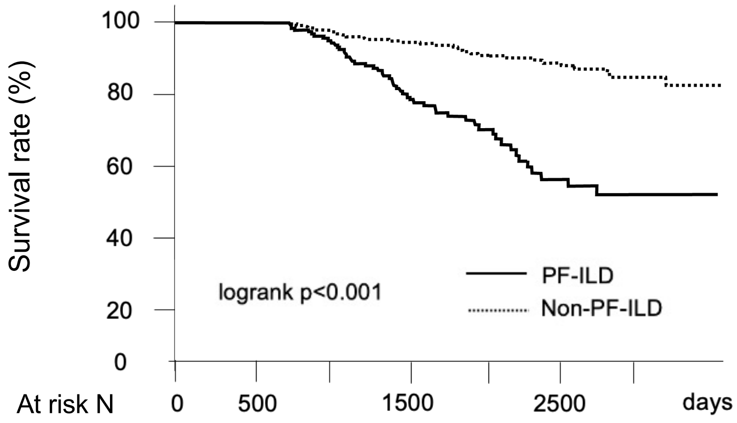

At risk N							
PF-ILD	167	167	144	95	50	28	15
Non-PF-ILD	383	375	324	239	164	107	50

Survival estimates were performed using the Kaplan–Meier method and compared by log-rank test between PF-ILD (n=167) and non-PF-ILD (n=383).
Abbreviations: PF-ILD, progressive fibrosing interstitial lung disease.
